# Fracture Patella

**Published:** 1889-10-12

**Authors:** 


### FRACTURED PATELLA.

"On the Treatment of Fractures of the Patella" is the title of an article by Mr. Owen Thomas, of Liverpool.* Fractured patella is ordinarily regarded as an accident difficult to treat, but Mr. Thomas says it is the easiest to restore and repair of all important fractures. Contrary, also, to the usual teaching, Mr. Thomas asserts that neither position nor nicety of adaptation are essential to a good result. A sketch is given of Mr. Thomas's method, which he calls "indirect," of fixing the patella. A bed knee-splint is fixed to the shoe below, and to the body above, broad bands round the middles of the tibia and of the femur, and under the popliteal space fixing the limb. By this, *non-interference* with the circulation around the patella is secured. The author details several cases treated by this method, the patients being able to walk and go about their business immediately after the fracture had been "put up," although some months is demanded for the cure. As regards the approximation of the fragments after recovery, the author states that "a perceptible space is a very exceptional result, the restoration and repair are usually perfect."

* *Provincial Medical Journal*, August 1, 1889.

Should this treatment prove as successful in other hands, Mr. Owen Thomas will deserve thanks for a great surgical advance.

